# Over-expression of Nav1.6 channels is associated with lymph node metastases in colorectal cancer

**DOI:** 10.1186/s12957-019-1715-4

**Published:** 2019-10-31

**Authors:** Shuiquan Lin, Yangbo Lv, Jianguang Xu, Xinglong Mao, Zhenhong Chen, Wuguang Lu

**Affiliations:** 1 Department of Anorectal Surgery, Quzhou People’s Hospital, Quzhou, 324000 Zhejiang People’s Republic of China; 2 Department of Digestive System, Quzhou People’s Hospital, Quzhou, 324000 Zhejiang People’s Republic of China; 3 Department of Gastrointestinal Surgery, Quzhou People’s Hospital, Quzhou, 324000 Zhejiang People’s Republic of China; 4 Affiliated Hospital of Integrated Traditional Chinese and Western Medicine, Nanjing University of Chinese Medicine, Nanjing, 210028 Jiangsu People’s Republic of China

**Keywords:** Colorectal cancer, Sodium channels, Lymph nodes

## Abstract

**Background and objectives:**

Lymph node metastasis is a key factor in predicting and determining the prognosis of patients with colorectal cancer (CRC). Sodium channels are highly expressed in a variety of tumors and are closely related to tumor development, metastasis, and invasion. We investigated the relationship between the expressions of different subtypes of Nav channels and lymph node metastasis of CRC.

**Methods:**

Real-time PCR (RT-qPCR) was carried out to measure the expressions of different sodium channel subtypes, chemokine receptors (CCR2, CCR4, CCR7), and lymphocyte infiltration-related biomarkers (CD3e, CD8a, IL-2RA) in CRC tissues from 97 patients. The expressions of Nav1.5 and Nav1.6 in surgically isolated lymph nodes were detected by immunohistochemistry. Correlation analysis between expressions of different genes and lymph node metastasis was performed by two-tailed *t* test.

**Results:**

Nav1.1 and Nav1.6 were highly expressed in CRC tissues and positively correlated with CRC lymph node metastasis. Nav1.6 was also highly expressed in metastatic lymph nodes. Further analysis showed that the high expression of Nav1.6 was closely related to the one of CCR2\CCR4 in tumor lymph node metastasis.

**Conclusions:**

These results suggested that Nav1.6 might be a novel marker for CRC lymph node metastasis.

## Background

Colorectal cancer (CRC) is the second leading cause of tumor-related death and the third most common malignant tumor worldwide, only secondary to lung cancer and breast cancer [1, 2]. The main cause of death from CRC is distant metastasis of the tumor. The distant metastasis of CRC leads to its advanced stage with low remission and survival rates in patients. Lymph node metastasis is the most common metastatic pathway of CRC [3] and is also a key factor in predicting and determining the prognosis of patients [4]. O’Connell et al. reviewed clinical and pathological data of more than 100,000 patients with CRC [2]. Results showed that the 5-year stage-specific survivals were 93.2% for stage I, 84.7% for stage IIa, 72.2% for stage IIb, 83.4% for stage IIIa, 64.1% for stage IIIb, 44.3% for stage IIIc, and 8.1% for stage IV [2] (AJCC sixth edition system). Leonard L et al.’s statistics showed that the 5-year survival rate was 65.4% in patients with lymph node metastasis and 94.4% in patients without lymph node metastasis [5]. Therefore, the pathological examination of lymph nodes in CRC patients is crucial for post-operative treatment and patient prognosis [4].

The early diagnosis of tumor lymph node metastasis depends on the detection of specific markers of lymphangiogenesis. VEGFR3 is a relatively specific receptor expressed on lymphatic endothelial cells and involved in the signal transduction of activation of lymphatic endothelial cells [6]. However, VEGFR3 is also expressed on porous capillaries in endocrine organs as well as on monocytes and megakaryocytes. The other two lymphatic endothelial growth factors, VEGF-C and VEGF-D, promote lymphangiogenesis by binding to their receptor VEGFR-3 and induce anastomoses between lymphatic vessels and lymphatic vessels [6]. Other potential lymph node metastasis markers such as lymphatic vessel endothelial hyaluronan receptor 1 (LYVE-1) expressed on lymphatic vessel endothelium is considered to be a specific marker of lymphatic vessels. LYVE-1 was overexpressed in colon tumors compared with in unaffected colon tissues [7]. However, these lymphatic vessel markers only indicate the lymphatic hyperplasia of the tumor rather than directly indicate lymph node metastasis, and thereby cannot be used as a clinical diagnostic indicator to assess patient prognosis. As a result, it is essential to search for more specific markers of lymph node metastasis.

Voltage-gated Na^+^ channels (VGSCs) are macro-molecular protein complexes embedded in cell membranes, which are composed of a channel-forming alpha subunit and one or more smaller beta subunits [8]. The VGSC α subunit family contains nine members, Nav1.1–Nav1.9, encoded by the *SCN1A–SCN11A* genes [8]. VGSCs are abundantly expressed in excitable cells such as neurons and cardiomyocytes, which are responsible for the generation of action potentials and the transmission of neural signals [8]. However, VGSCs have been shown to be expressed in tumor cells from a variety of cancers, such as breast cancer, cervical cancer, colon cancer, melanoma, neuroblastoma, non-small cell lung cancer, ovarian cancer, and prostate cancer [9]. Abnormally high expression of VGSC alpha subunits in cancer cells promotes migration and invasion of tumor cells [9]. Nav1.1, Nav1.2, Nav1.3, Nav1.4, and Nav1.9 are expressed in ovarian cancer, non-small cell lung cancer, and prostate cancer [10]. The Nav1.7 alpha subunit promotes gastric cancer progression through MET transcriptional regulator-mediated upregulation of sodium-hydrogen antiporter 1 [10]. Other studies have shown that the expression of the Nav1.5 alpha subunit is closely related to a poor prognosis in breast cancer [11], non-small cell lung cancer [12], ovarian cancer [13], and prostate cancer [14]. The Nav1.5 alpha subunit is also a key regulator of the transcriptional regulatory network of genes that control CRC cell invasion [15]. The Nav1.5 alpha subunit controls colon cancer metastasis via regulation of Wnt signaling, cell migration, ectodermal development, steroid metabolism, and cell cycle-dependent protein expression [15]. Other studies have shown that Nav1.5 upregulates CRC-inducible gene expression through the MAPK signaling pathway, therefore promoting colon cancer metastasis [16].

Nav1.6 is expressed in breast cancer, cervical cancer, lymphoma, melanoma, mesothelioma, non-small cell lung cancer, prostate cancer, and small cell lung cancer [9]. Furthermore, the over-expression of Nav1.6 can promote the metastasis and invasion of cervical cancer [9, 17]. Similarly, our study found that Nav1.6 was highly expressed in CRC tissues and positively correlated with lymph node metastasis. Additionally, in the surgically removed lymph nodes, we found that Nav1.6, not Nav1.5, was highly expressed in metastatic lymph nodes.

Lymph node metastasis requires tumor cells to flow in or settle in the marginal sinus of the lymph nodes. Tumor-associated macrophages (TAMs) or M2 macrophages play an important role in the development, metastasis, and prognosis of a variety of malignant, metastatic tumors [18]. For example, in local lymph node metastasis of oral squamous cell carcinoma, M2 macrophage infiltration into the marginal sinus of the lymph node increased with malignancy [19]. Chemokine receptor 2 (CCR2)-positive monocytes and macrophages are classified as TAMs, both of which are regulated by the CCR2/CCL2 axis [20]. Another chemokine receptor CCR4 is an important chemokine receptor that regulates immune homeostasis and is thought to be involved in the progression of hematological malignancies [21]. CCR4 expression is positively correlated with HER2 expression, tumor recurrence, and lymph node, lung, and bone metastasis of breast cancer [21]. Autocrine and paracrine loops between cancer cells and macrophages promote lymph node metastasis via CCR4/CCL22 axis in head and neck squamous cell carcinoma [22]. Our result indicated that CCR2 and CCR4 were also highly expressed in CRC tissues and positively correlated with lymph node metastasis and over-expression of Nav1.6. This study suggests that Nav1.6 could be used as a potential biomarker for lymph node metastasis in patients with CRC. And the CCL2-CCR2 and CCL22-CCR4 axis may be the mechanism of high expression of Nav1.6 promoting lymph node metastasis of CRC.

## Materials and methods

### Biological samples

The enrolled cases in this study were patients with tumor lesions larger than 2 cm, who were diagnosed as stage II–III colon cancer (AJCC eighth edition system) without radiotherapy and chemotherapy. After laparoscopic surgery in CRC patients, the acquired specimens were sampled immediately in vitro. A 0.5 × 0.5-cm piece of CRC tumor tissue from the intestinal lumen surface was isolated as the tumor sample for further study. Meanwhile, 0.5 × 0.5 cm of the intestinal wall tissue (mainly mucosa and submucosa) 5 cm far from the edge of the tumor was isolated as a normal tissue control. All acutely isolated clinical samples were stored in a − 86 °C freezer within 2 min. The remaining tumor tissue was immediately preserved in 4% formalin for subsequent HE and immunochemistry (IHC) staining.

### Real-time quantitative PCR

The total RNA from tumor and normal tissues from 97 CRC patients were prepared by using TRIzol reagent (Invitrogen). RNA (1 μg) was reverse-transcribed into cDNA using the Maxima First Strand cDNA Synthesis Kit according to the standard manufacturer’s protocol (Fermentas, USA). Real-time quantitative PCR (RT-qPCR) was performed by using the 2× Maxima SYBR Green/ROX qPCR Master Mix kit with ABI 7500 according to the standard manufacturer’s protocol (Fermentas, USA). Data were analyzed using the ΔΔCT relative quantification method. Primer sequences and corresponding genes are shown in Table 1.

**Table 1:** Quantitative PCR primers used in this study. Designed with NCBI primer blast. Product length, 100–200 bp; annealing temperatures, 57–60 °C

Gene	Genebank no.	Sequence
hNav1.1	NM_001165963.1	ACATTACGTGCTGCTGGGAA CCAAGGTGGCCTGATTCTGT
hNav1.2	NM_001040142.1	TGACAGAGTTTGTGGACCTGG GCGCAAACACGCTTAGACAG
hNav1.3	NM_001081676.1	TTCAGTTTCAGAGGTCGGGC TTTCTGTGGTGGTGCCGTTA
hNav1.4	NM_000334.4	CGGCTGGTGTGTCATACTGT TAGGCCAACAGAATGCCCTG
hNav1.5	NM_000335.4	TTTGTGGACCTGGGCAATGT GGCAGAAGACTGTGAGGACC
hNav1.6	NM_001177984.2	GGAAGCCTGTCTTGGTGTGT TTTGCGAGAGCCCCTTTGAT
hNav1.7	NM_002977.3	GACGCAGCAGTAACATCAGC TTGATTGGTCGTGCCCTCTG
hNav1.8	NM_001293306.2	TCTCTGTGCCCATTGCTGAG CAGCTGCTCCTGTCCTTTGG
hNav1.9	NM_001287223.1	GTCTGGGAAAGACCAGCCTC CAGTGCTCTCTGCCTTTGGA
hCCR-2	NM_001123041.2	AGTCAACTGGACCAAGCCAC TGTGAAAAAGGCTTCTGAACTTCT
hCCR-3	NM_001837.3	CAGGAGTGGTGACGCCTAAG ACCAGCTTCTCATCTGCTGAA
hCCR-4	NM_005508.4	AAAGCAAGCTGCTTCTGGTTG AGGAAGAGCTCCCCAAATGC
hCCR-7	NM_001301714.1	CATGGACCTGGGTATGCCTG AAAGTTCCGCACGTCCTTCT
hBcl-2	NM_000633.2	TGAACTGGGGGAGGATTGTG CGTACAGTTCCACAAAGGCA
hTGFbetaR1	NM_001130916.2	TCCAACTACTGGTTTACCATTGC TTCTTCTCCCCGCCACTTTC
hCD3e	NM_000733.3	GGCCTGAATCAGAGACGCAT TAGCCCAGGAAACAGGGAGT
hP53	NM_000546.5	ACCTATGGAAACTACTTCCTGAAA CTGGCATTCTGGGAGCTTCA
hCD8a	NM_001145873	TGAGTTCTCTTCTCCCCTACGA GCCGTACTGAGGCAGAAACG
hIL2RA	NM_000417.2	CAGTGCGTCCAGGGATACAG GACGAGGCAGGAAGTCTCAC
hIFNGR	NM_000416.2	CAGCATGGCTCTCCTCTTTCTC TAGTTGGTGTAGGCACTGAGG
hGAPDH	NM_001256799.2	ACATCAAGAAGGTGGTGAAGCA GTCAAAGGTGGAGGAGTGGGT

### Immunochemistry staining

Specimens were cut into 4-μm sections and adhered to glass slides. For HE staining, slides were deparaffinized and hydrated to water and then stained in hematoxylin solution, then differentiated sections with acid alcohol for two times and washed sections with ammonia solution. After being washed in a slow running tap water and stained in eosin, slides were dehydrated and mounted for imaging. The nucleus showed blue and the cytoplasm showed red. For immunochemistry staining, an anti-Nav1.5 antibody (ab56240 Abcam) and an anti-Nav1.6 antibody (ab6516 Abcam) were used to detect the expression of Nav1.5 and Nav1.6 in the lymph node, respectively. After the retrieval of antigens and an endogenous peroxidase block, slides were incubated with primary antibody overnight at 4 °C. After the incubation of the secondary antibody, add a freshly prepared DAB chromogenic reagent to mark the tissue and counterstain in the nucleus. Then dehydrated slides in gradient ethanol and then washed slides in xylene and mount with resin mounting medium. The nucleus stained with hematoxylin is blue. The positive cells developed by DAB reagent have a brown-yellow nucleus. For the negative control, samples were analyzed using a phosphate-buffered saline instead of the primary antibodies.

### Data analysis

Statistical significance was analyzed using SPSS 20 software. Multiple comparisons were performed using a two-tailed *t* test. Differences were considered significant when *P* < 0.05. The asterisks in Figs. 2, 3, and 4 indicate significant differences (*P < 0.05, **P < 0.01, and ***P < 0.001).

## Results

### CRC sampling and lymph node metastasis detection

**Fig. 1:**
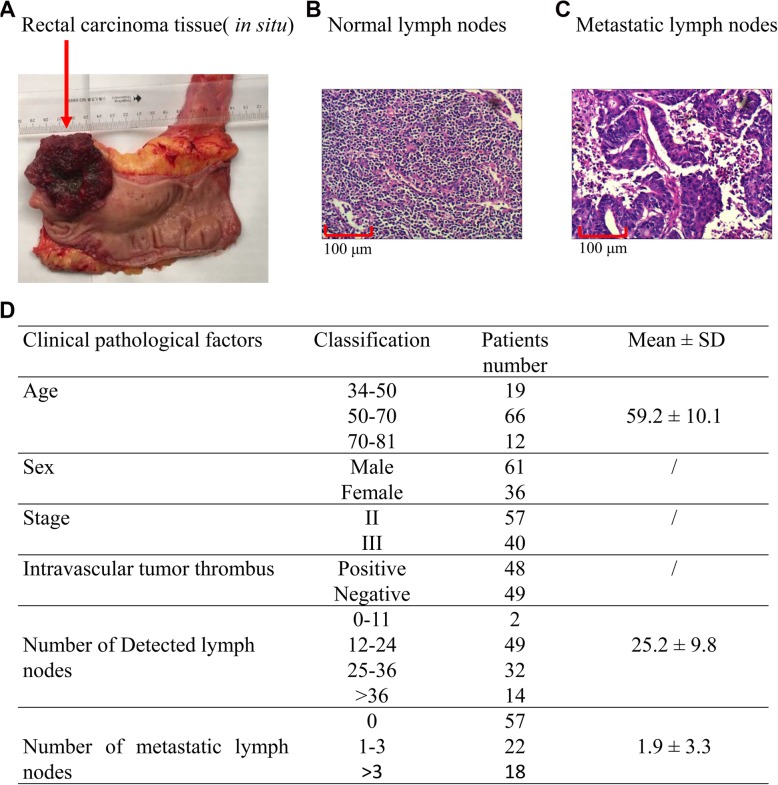
CRC sampling and lymph node metastasis detection. **a** The tissue removed via surgery for CRC radical resection, including the intestine approximately 25 cm from the anus, the corresponding total mesorectal and sigmoid mesenteric tissue, the inferior mesenteric artery, and the surrounding lymph node tissue. The tumor was round with an ulcerated diameter of approximately 3.5–4 cm (marked with red arrow). **b** is a 10 × 10-fold hematoxylin and eosin (HE) staining map of a non-metastatic lymph node, and its cellular structure and cell size are not significantly abnormal. **c** represents an HE staining map of a metastatic lymph node. **d** is a collective statistical analysis of lymph node metastasis and clinicopathological parameters from 97 CRC patients. For colon cancer, the resection range includes colon-centered, 10-cm upper and lower colon tissue and the corresponding mesenteric and mesenteric root tissues; for rectal cancer, the resected tissue includes tumor-centered proximal 10 cm, down 2–5 cm (depending on the location of the tumor from the anus and whether the anus is retained) and the corresponding mesenteric and mesenteric root tissues. All lymph nodes in the tissue were searched postoperatively and their metastases were counted

Figure 1a shows the tissue removed via surgery of colon cancer radical resection, including the intestine approximately 25 cm from the anus, the corresponding total mesorectal and sigmoid mesenteric tissue, the inferior mesenteric artery, and the surrounding lymph node tissue. The tumor was round with an ulcerated diameter of approximately 1.5 cm, and the lower edge was 1 cm from the dentate line. A 10 × 10-fold hematoxylin and eosin (HE) staining map indicated that the cellular structure and cell size of a non-metastatic lymph node were normal. In the metastatic lymph node, the cancer tissue infiltrated the metastatic lymph node and arranged in a glandular tube (Fig. 1b, c). Figure 1d is a collective statistical analysis of lymph node metastasis from CRC patients. We collected 97 first-episode patients with CRC without preoperative chemo-radiotherapy. Among the 97 patients aging from 34 to 81 years old, with a male to female ratio of 61:36, the stage II/III ratio is 57:40, with/without an intravascular tumor thrombus ratio of 48:49, and with/without a perineural invasion ratio of 34:63. The total number of detected lymph nodes was 25.2 ± 9.8, of which 41.2% were lymph node metastasis and the total number of positive lymph nodes was 1.9 ± 3.3.

### Various sodium channel subtypes were highly expressed in human CRC tissues

**Fig. 2:**
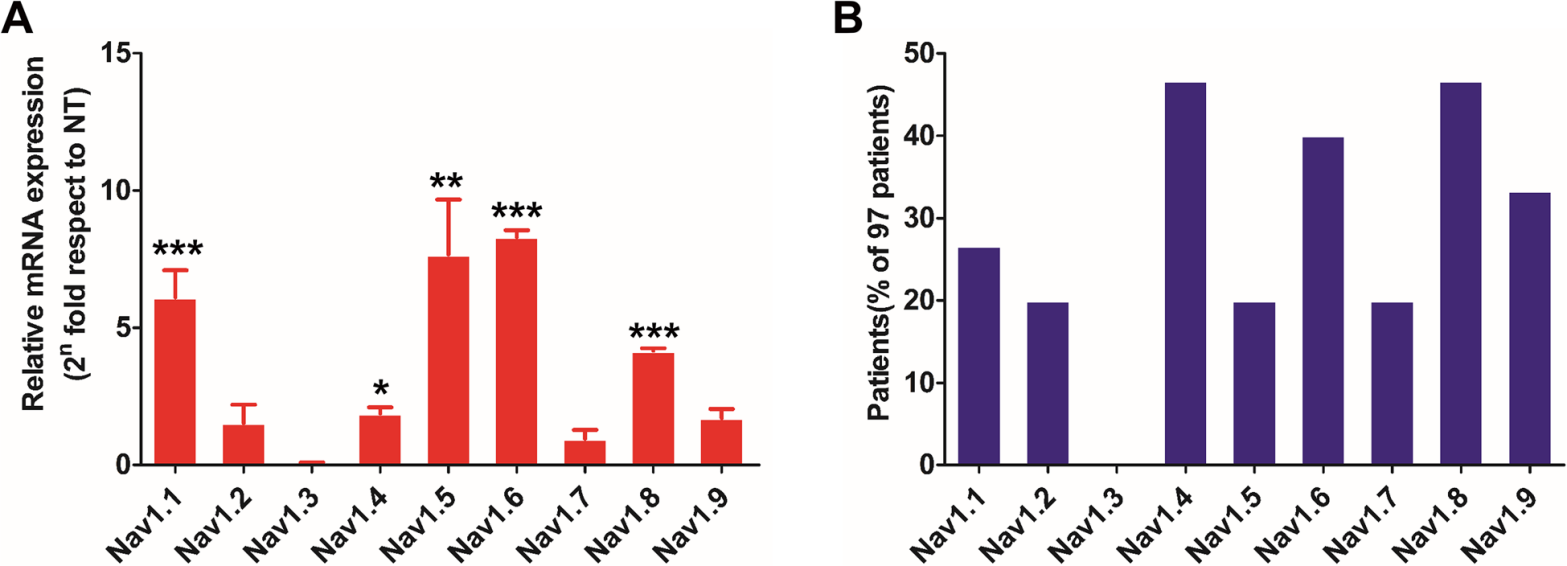
Analysis of mRNA expression of voltage-gated Na^+^ channel (VGSC) subtypes in colorectal cancer (CRC) tissues. Differences between the VGSC expression levels were expressed as fold change relative to normal tissue using the 2^–∆∆CT^ method. **a** Relative mRNA expression of VGSC subtypes in 97 CRC tissues. **b** Proportion of patients with high expression levels of different subtypes of VGSCs in 97 CRC patient samples

From the cancer tissues of 97 CRC patients, the total RNA was extracted and the expression levels of each VGSC were analyzed using qPCR. The expression levels in the normal tissues of patients were taken as a reference control. The patients with tissue VGSC expression levels twice as high as those of normal tissue were included in this metric. The results showed that Nav1.1, Nav1.2, Nav1.4, Nav1.5, Nav1.6, Nav1.8, and Nav1.9 were highly expressed in CRC tissues (Fig. 2a). In particular, the expression levels of Nav1.5, Nav1.6, and Nav1.8 increased more than eight times compared to normal tissues (Fig. 2a). Furthermore, patients with a high expression of Nav1.4, Nav1.6, and Nav1.8 accounted for more than 30% of the total number of CRC patients (Fig. 2b).

### Over-expression of Nav1.6 positively correlated with lymph node metastasis in CRC

**Fig. 3:**
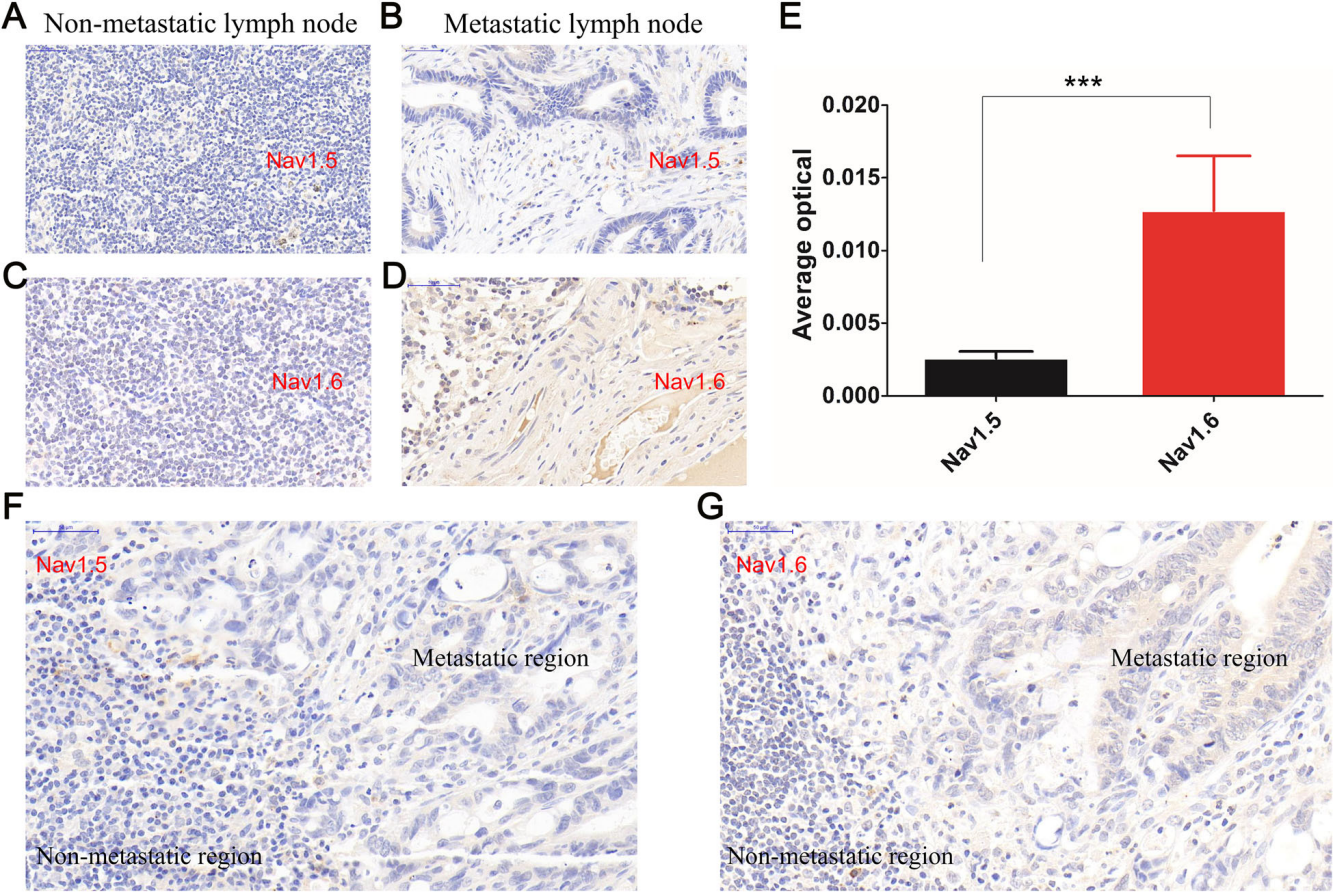
Immunohistochemistry for Nav1.5 and Nav1.6 in surgically isolated lymph nodes. **a**, **b** Nav1.5 is lowly expressed in both non-metastatic and metastatic lymph nodes (magnification 10 × 40). Nav1.6 is lowly expressed in non-metastatic lymph nodes (**c**) but highly expressed in metastatic lymph nodes (**d**) (magnification, 10 × 40). **e** Comparison of the average optical density of Nav1.5- and Nav1.6-positive regions in metastatic lymph nodes (data from five patients). At least 3200-fold fields of view were randomly selected for each slice in each group for screenshots. Image-Pro Plus 6.0 software was used to select the same brown color as the unified standard for judging all photos. Each photo was analyzed to obtain the cumulative optical density (IOD) of each photo and the area of the tissue (AREA) and obtain the average optical density value (average optical, AO value), AO = IOD/AREA, the greater the AO value, the higher the positive expression level. **f**, **g** Expression of Nav1.5 and Nav1.6 in different regions of lymph node. The lower left area of the figure was the non-tumor metastatic region and the right area was metastatic region. There was no significant difference in the expression of Nav1.5 between these two regions while Nav1.6 is lowly expressed in the non-metastatic region but highly expressed in the metastatic region

The correlation between the upregulation of VGSCs by each subtype and lymph node metastasis was further analyzed using the *t* test. Nav1.1 and Nav1.6 expression levels were significantly higher in CRC tissues with lymph node metastasis than those without metastasis, whereas other elevated expression levels of VGSC subtypes did not show significant correlation with CRC lymph node metastasis (Table 2). The immunochemistry staining result indicated that the non-metastatic lymph nodes are structurally intact and the lymphocytes are evenly distributed (Fig. 3a, c). In metastatic lymph nodes, multiple metastatic adenocarcinoma tissues were found (Fig. 3b, d). Nav1.5 was expressed at low levels in the interstitial tissues of non-metastatic lymph nodes and in the cytoplasm of metastatic lymph node glands (Fig. 3a, b). In contrast, Nav1.6 is highly expressed in the metastatic lymph nodes of the glandular epithelial cytoplasm (Fig. 3c, d). The analysis of the mean optical density values of the Nav1.5- and Nav1.6-positive regions in metastatic lymph nodes revealed that the expression of Nav1.6 was much higher than that of Nav1.5 (Fig. 3e). In the lymph node, there was a significant structural difference between the metastatic region and non-metastatic region. Although there was no significant difference in the expression of Nav1.5 between these two regions, the expression of Nav1.6 in the metastatic region was much higher than that in the non-metastatic region (Fig. 3f, g).

**Table 2:** Two-tailed *t* test analysis of the correlation between the upregulation of different sodium channel subtypes and lymph node metastasis in CRC tissues

Nav channel	Lymph node metastasis CRC	Non-lymph node metastasis CRC	*T*	*P*
Nav 1.1	3.19 ± 2.91	2.05 ± 1.96	2.304	*0.023*
Nav 1.2	2.07 ± 2.37	1.97 ± 1.96	0.213	0.832
Nav 1.3	0.34 ± 0.45	0.41 ± 0.52	− 0.682	0.497
Nav 1.4	3.08 ± 2.90	2.23 ± 2.21	1.630	0.106
Nav 1.5	1.64 ± 1.34	1.17 ± 1.186	1.811	0.073
Nav 1.6	3.63 ± 3.21	2.13 ± 1.96	2.534	*0.014*
Nav 1.7	1.80 ± 1.76	2.13 ± 2.48	− 0.683	0.496
Nav 1.8	3.11 ± 2.72	2.59 ± 1.94	1.098	0.275
Nav 1.9	1.66 ± 1.48	1.86 ± 1.70	− 0.603	0.548

The independent sample *t* test in SPSS20.0 was used to determine whether lymph node metastasis was a grouping variable, and the expression of the sodium ion channel subtype was a test variable. When the sig value of the Levene test is greater than 0.05, the *T* value and the *P* value are obtained by assuming the variance is equal. When the sig value of the Levene test is less than 0.05, the *T* value and the *P* value are obtained by assuming that the variances are not equal. An obtained *P* value < 0.05 is indicated in italic

Since the presence of lymphovascular invasion is also a key parameter predicting patients’ prognosis in various types of cancer, we further analyzed the correlation between the upregulation of different Nav channels and lymphovascular invasion. The two-tailed *t* test analysis results suggested that the over-expression of Nav1.6 and Nav1.7 is positively related to lymphovascular invasion in CRC (Table 3).

**Table 3:** Two-tailed *t* test analysis of the correlation between the upregulation of different sodium channel subtypes and lymphovascular invasion in CRC tissues

Nav subtype	Lymphovascular invasion	Non-lymphovascular invasion	*T*	*P*
Nav1.1	2.70 ± 2.90	2.31 ± 1.98	0.791	0.431
Nav1.2	1.58 ± 1.38	2.32 ± 2.48	− 1.874	0.064
Nav1.3	0.40 ± 0.51	0.37 ± 0.49	0.232	0.817
Nav1.4	2.79 ± 2.84	2.36 ± 2.25	0.831	0.408
Nav1.5	1.41 ± 1.38	1.30 ± 1.18	0.399	0.691
Nav1.6	3.83 ± 3.15	1.86 ± 1.68	3.639	*0.001*
Nav1.7	2.60 ± 2.58	1.57 ± 1.85	2.287	*0.024*
Nav1.8	3.15 ± 2.68	2.50 ± 1.88	1.405	0.163
Nav1.9	1.41 ± 1.30	2.06 ± 1.78	− 1.959	0.053

The independent sample *t* test in SPSS20.0 was used to determine whether lymph node metastasis was a grouping variable, and the expression of the sodium ion channel subtype was a test variable. When the sig value of the Levene test is greater than 0.05, the *T* value and the *P* value are obtained by assuming the variance is equal. When the sig value of the Levene test is less than 0.05, the *T* value and the *P* value are obtained by assuming that the variances are not equal. An obtained *P* value of < 0.05 is indicated in italic

### Over-expression of Nav1.6 positively correlated with the expression of CCR2 and CCR4

**Fig. 4:**
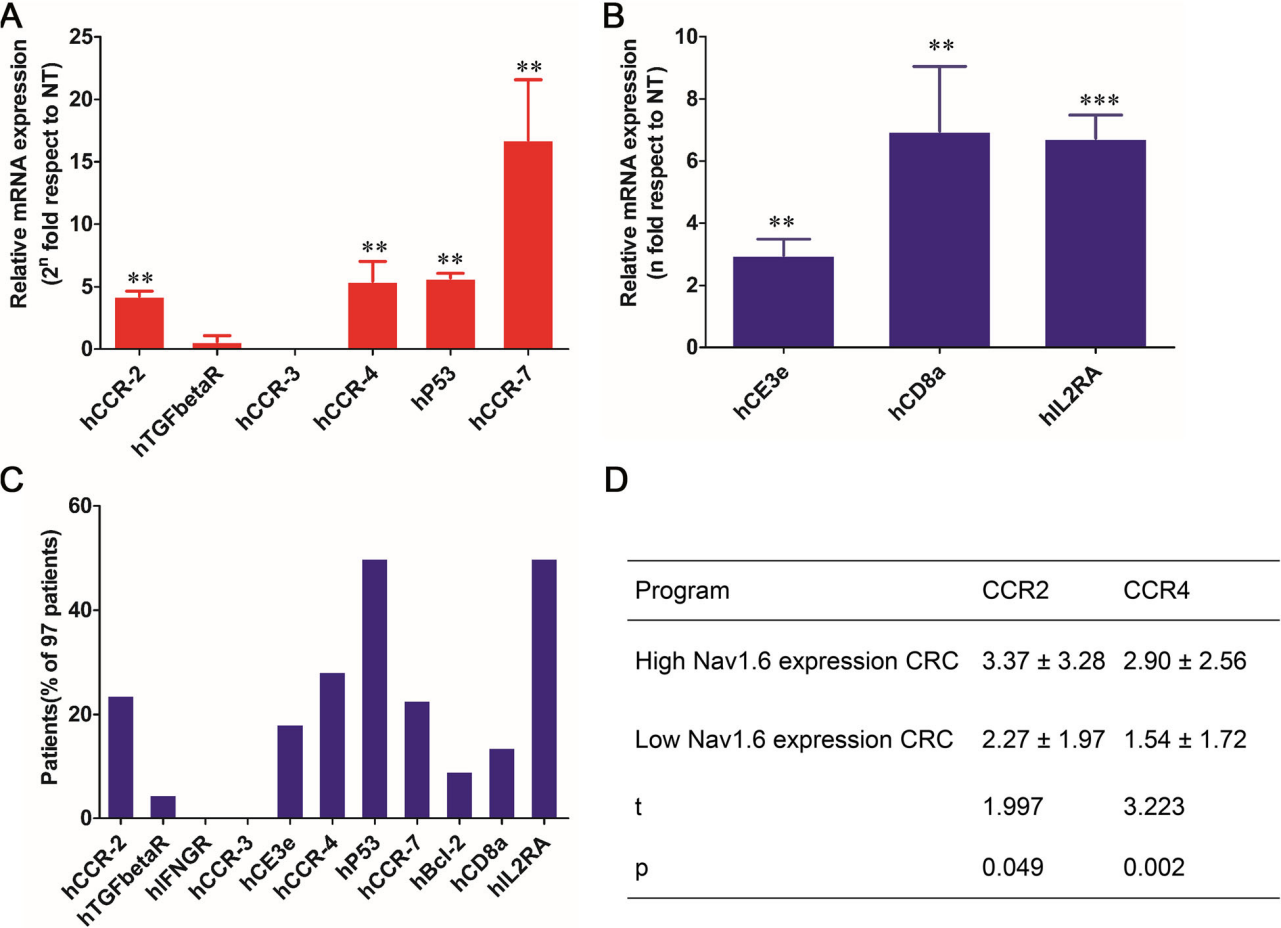
Association of known markers of lymph node metastasis and infiltration with Nav1.6. **a**, **b** Quantitative PCR analysis of mRNA expression levels for lymphocyte infiltration- and lymph node metastasis-related molecular biomarkers in 97 CRC tissues. **c** Proportion of patients with a high expression of lymphocyte infiltration- and lymph node metastasis-related molecular biomarkers in 97 CRC patients. **d** Two-tailed *t* test analysis of the correlation between the upregulation of CCR2 and CCR4 and lymph node metastasis in CRC tissues. The independent sample *t* test in SPSS20.0 was used to determine whether nav1.6 was highly expressed as a grouping variable and CCR2\CCR4 as a test variable. When the sig value of the Levene test is greater than 0.05, the *T* value and the *P* value are obtained by assuming the variance is equal. When the sig value of the Levene test is less than 0.05, the *T* value and the *P* value are obtained by assuming that the variances are not equal

The biomarkers of lymphatic invasion and lymph node metastasis in clinical specimens were analyzed using RT-qPCR. The results showed that the chemokine receptor biomarkers CCR2, CCR4, and CCR7 were highly expressed in lymph node metastatic tissues compared with normal tissues (Fig. 4a). In addition, lymphocyte infiltration-related biomarkers CD3e, CD8a, and IL-2RA were highly expressed in CRC tissues (Fig. 4b). Further analysis found that the proportion of patients with high expression of CCR2, CCR4, and CCR7 in CRC patients was over 20%, whereas those with high expression of IL-2RA was as high as 47% (Fig. 4c). Patients with high expression of CD3e and CD8a were less than 20% (Fig. 4c). However, the further two-tailed *t* test analysis suggested that only the over-expression of CCR2 and CCR4 shows significant correlation with the high expression of Nav1.6 (Fig. 4d).

### Over-expression of CCR2 and CCR4 positively correlated with lymph node metastasis in CRC

Based on the above RT-qPCR quantification results in combination with the patients’ diagnosis and treatment information, the correlation between lymph node metastasis and lymphocyte invasion and its relationship with the upregulation of metastasis-related molecular biomarkers were further analyzed using *t* tests. The results illustrated higher hCCR-2 and hCCR-4 expression in tumor tissues with lymph node metastasis than in those without lymph node metastasis (Table 4). This result suggested that high hCCR-2 and hCCR-4 expressions were significantly associated with lymph node metastasis in CRC.

**Table 4:** Analysis of the correlation between the upregulation of lymph node metastasis-related molecular markers and lymph node metastasis in colorectal cancer (CRC) tissues

Gene	Lymph node metastasis CRC	Non-Lymph node metastasis CRC	*T*	*P*
hCCR2	4.21 ± 3.67	2.24 ± 2.12	2.949	*0.005*
hTGFbetaR	0.94 ± 0.54	0.91 ± 0.55	0.215	0.830
hCD3e	2.65 ± 1.52	2.52 ± 2.12	0.304	0.762
hCCR4	3.40 ± 3.50	2.03 ± 1.85	2.168	*0.035*
hP53	2.23 ± 1.5 2	2.33 ± 1.59	− 0.305	0.761
hCCR7	6.21 ± 5.56	6.44 ± 6.65	− 0.172	0.864
hBcl2	2.41 ± 1.45	2.14 ± 1.50	0.883	0.380
h1L2Ra	6.45 ± 5.13	4.68 ± 3.65	1.973	0.051
hCD8a	11.55 ± 8.95	11.21 ± 12.49	0.144	0.886

Two-tailed *t* test in SPSS20.0 was used to determine whether lymph node metastasis was a group variable, and the expression of each immune factor was a test variable. When the sig value of the Levene test is greater than 0.05, the *T* value and the *P* value are obtained by assuming the variance is equal. When the sig value of the Levene test is less than 0.05, the *T* value and the *P* value are obtained by assuming that the variances are not equal. *P* < 0.05 is marked in italic

## Discussion

The pathological examination of lymph nodes in patients with CRC is critical for post-operative treatment and prognosis prediction [4]. Early diagnosis of tumor lymph node metastasis relies on the use of specific sensitive lymphatic biomarkers and advanced techniques to make an accurate diagnosis of lymph angiogenesis and lymph node metastasis. However, the existing lymphatic metastasis markers are mostly lymphatic endothelial markers, which are not suitable for clinical diagnosis and evaluation of patient prognosis.

Abnormal expression of various sodium channel alpha subunits is closely related to tumor invasion and metastasis, but the relationship between sodium channel over-expression and tumor lymph node metastasis is still unclear [9]. Previous studies have shown that the Nav1.5 alpha subunit plays a key role in the transcriptional regulatory network that controls colon cancer cell invasion [15]. Our results showed that Nav1.1, Nav1.2, Nav1.4, Nav1.5, Nav1.6, Nav1.8, and Nav1.9 were highly expressed in CRC tissues. However, only a high expression of Nav1.1 and Nav1.6 rather than Nav1.5 was positively correlated with lymph node metastasis. Further immunohistochemistry shows that Nav1.5 and Nav1.6 were both lowly expressed in non-metastatic lymph nodes, while Nav1.6 was highly expressed in metastatic lymph nodes. These results suggested that a high expression of Nav1.1 and Nav1.6 may promote lymph node metastasis in colorectal cancer. Nav1.1 is mainly expressed in the central nervous system, especially the brain, whereas Nav1.6 is mainly expressed in the peripheral nervous system and epithelial cells, especially in the intestinal nerve center and intestinal epithelial cells [8]. Therefore, Nav1.6 is more suitable for in-depth study as a potential CRC lymph node metastasis marker.

CCR2, CCR4, and their respective ligand-mediated pathways, CCR2\CCL2 axis and CCR4\CCL22 axis, are considered to be important mechanisms of lymph node metastasis [21, 23]. In this study, we found that CCR2 and CCR4 were highly expressed in Nav1.6 high-expression CRC tissues and were positively correlated with lymph node metastasis. CCR2\CCL2 axis and CCR4\CCL22 axis may involve in the over-expression of Nav1.6 promoting lymph node metastasis. However, whether the upregulation of Nav1.6 expression promotes lymph node metastasis through CCR2\CCL2 axis and CCR4\CCL22 axis needs further study.

Tumor-infiltrating lymphocytes (TILs) are predictors of lymph node metastasis in many cancers such as early gastric cancers, melanoma, and rectal cancers [24–26]. High TILs were significantly associated with an absence of lymph node metastasis [24]. A high frequency of CD8-positive lymphocyte infiltration correlates with the lack of lymph node involvement in early rectal cancer [26]. Among the 97 patients, the proportion of patients with a high frequency of CD8-positive lymphocyte infiltration was about 15%. This result indicated that these patients with a high frequency of CD8-positive lymphocyte infiltration may have a less probability of lymph node metastasis in the future.

## Conclusion

Our study suggests that Nav1.6 may be a potential biomarker for lymph node metastasis in patients with CRC.

## Data Availability

The datasets during and/or analyzed during the current study are available from the corresponding author on reasonable request.
